# Immune-Related Adverse Events in Patients with Melanoma Treated with B-RAF/MEK Target Therapy: Occurrence and Circulating Immune Cell Analysis

**DOI:** 10.3390/cancers18071072

**Published:** 2026-03-26

**Authors:** Alessia Capone, Maria Luigia Carbone, Simona Mastroeni, Francesca Romana Di Pietro, Sofia Verkhovskaia, Roberto Morese, Nidia Melo, Stefania D’Atri, Federica De Galitiis, Rosa Falcone, Cristina Fortes, Elisabetta Volpe, Cristina Maria Failla

**Affiliations:** 1 Molecular Neuroimmunology Laboratory, IRCCS Santa Lucia Foundation, 00179 Rome, Italy; a.caponepost@gmail.com (A.C.); e.volpe@hsantalucia.it (E.V.); 2 Experimental Immunology Laboratory, IDI-IRCCS, 00167 Rome, Italy; marialuigia.carbone@idi.it (M.L.C.); c.failla@idi.it (C.M.F.); 3 Clinical Trial Center, IDI-IRCCS, 00167 Rome, Italy; 4 National Centre for Disease Prevention and Health Promotion, Italian National Institute of Health, 00161 Rome, Italy; simona.mastroeni@iss.it; 5 Department of Oncology and Dermato Oncology, IDI-IRCCS, 00167 Rome, Italy; f.dipietro@idi.it (F.R.D.P.); s.verkhovskaia@idi.it (S.V.); r.morese@idi.it (R.M.); f.degalitiis@idi.it (F.D.G.); 6 Clinical Epidemiology Unit, IDI-IRCCS, 00167 Rome, Italy; n.salcedo@idi.it (N.M.); c.fortesepi@gmail.com (C.F.); 7 Molecular Oncology Laboratory, IDI-IRCCS, 00167 Rome, Italy; s.datri@idi.it

**Keywords:** immune-related adverse events, melanoma, target therapy

## Abstract

Background/Objectives: BRAF and/or MEK inhibitors are widely used for patients with BRAF-mutated melanoma, but no biomarkers of response or resistance are currently available. Besides adverse events in different organs, target therapy with BRAF and/or MEK inhibitors may induce the onset of immune-related adverse events (irAEs) that have been considered as possible biomarkers of good prognosis in patients with melanoma. Methods: To investigate this aspect, we analyzed the occurrence of irAEs in a cohort of 158 patients treated with BRAF and MEK inhibitors. We also analyzed by flow cytometry the subsets of circulating immune cells in the patients who developed irAEs and matched controls. Results: We found that irAEs occurred in 3 out of 101 patients (3%) who experienced adverse events. These three patients did not exhibit any specific clinical features or circulating immune cell subtypes that could be associated with a positive response to treatment. However, the onset of toxicity in the entire patient cohort was associated with longer progression-free survival. Notably, the frequency of circulating follicular helper T cells increased in all examined patients during the first two months of treatment. Conclusions: The small sample size prevents us from determining whether irAEs are effectively caused by BRAF/MEK inhibitors or if they are a random event. Additionally, we cannot conclude whether irAEs are related to a better outcome. Nevertheless, we note that BRAF/MEK inhibition may alter the composition of circulating immune cells in melanoma patients. This aspect should be investigated further before proposing combinations of target therapies and immunotherapies.

## Simple Summary

Target therapy with anti-BRAF and anti-MEK inhibitors is currently used for BRAF-mutated melanomas, but biomarkers of response or resistance have not yet been identified. It was previously reported that this target therapy has an immunomodulator activity and the onset of immune-related adverse events (irAEs) could represent a biomarker of response to therapy. Therefore, we analyzed both the occurrence of irAEs in a 158-patient cohort and the modulation of circulating immune cell subtypes in patients treated with anti-BRAF/anti-MEK inhibitors. We found a 3% occurrence of irAEs, too low an occurrence to consider them as response biomarkers. However, the onset of toxicity correlated with a longer progression-free survival. We also found an increment in the frequency of circulating follicular helper T lymphocytes in the first two months of treatment. The modulation of circulating immune cells by target therapy should be more deeply analyzed before the proposal of combined target and immunotherapy approaches.

## 1. Introduction

Cutaneous melanoma is the most hazardous skin cancer, and its incidence has been continuing to increase in the last five decades [1]. Fortunately, melanoma mortality is decreasing annually. This is mainly due to early screening campaigns, although more effective therapies, including immune checkpoint blockade and target therapy with combined anti-B-Raf protein (BRAF) and anti-mitogen-activated extracellular signal-regulated kinase (MEK) inhibitors (iBRAF/iMEK), also play a role. Usage of BRAF inhibitors derives from the recognition of mutation in the *BRAF* gene and from the induced signaling pathways [2]. *BRAF* mutations are among the most frequent mutations in human cancer, and they are present in approximately 50% of cutaneous melanomas. The majority of *BRAF* mutations in melanoma are represented by a substitution of valine at codon 600 with a glutamic acid (V600E). This mutation results in tumor hyperproliferation. Monotherapy with iBRAF is restricted because of the early development of acquired resistance due to multiple mechanisms such as *BRAF* amplification or downstream activating mutations in the MAPK pathway [3]. Currently, combination therapies with iBRAF/iMEK are used. Combination therapies have demonstrated superiority over monotherapy (iBRAF) in terms of response rate and survival. The phase III COMBI-d and COMBI-v trials showed that patients who have unresectable or metastatic melanoma with a BRAF V600E or V600K mutation and were treated with iBRAF/iMEK had prolonged progression-free survival (PFS) and overall survival (OS) compared with monotherapy treatment (dabrafenib and vemurafenib, respectively) [4,5]. Similarly, the COLUMBUS trial demonstrated that encorafenib plus binimetinib provided superior efficacy compared with iBRAF vemurafenib alone [6]. However, even with combined treatments, only 60% of patients achieved a five-year OS because of relapse and acquired resistance to therapy [7]. A subset of patients (15–21%) experienced a long-lasting response, particularly patients with low-volume disease and normal levels of lactate dehydrogenase (LDH) [7,8]. Today, no clinical or biological biomarkers have been developed that could predict response or resistance, either primary or secondary, to iBRAF/iMEK.

Toxicities induced by iBRAF/iMEK are not preventable, but they are generally manageable with dose reduction and seldom require treatment discontinuation (17% of patients) [8]. The most frequent adverse events (AEs) are pyrexia (41.7%), gastrointestinal symptoms (37.5%) and cutaneous manifestations (36.7%) [7,8,9]. AE incidence varies with the different combination of iBRAF and iMEK agents and may also depend on the patient specific concomitant therapies at baseline [10]. For example, cutaneous toxicity, including rash, pruritus, and photosensitivity, was higher in the vemurafenib plus cobimetinib combination [11], and cardiological toxicity significantly correlated with interactions of the iBRAF/iMEK treatment with concomitant drugs taken by the patients for preexisting comorbidities, such as Ace inhibitors, diuretics, sartans, or calcium antagonists [10].

Adjuvant therapy with iBRAF/iMEK dabrafenib plus trametinib was reported to increase the relapse free survival over placebo in patients with stage III BRAF V600-mutated melanomas, as demonstrated by the phase III COMBI-AD trial [12]. In this case, treatment resulted in manageable toxicity, less frequent in the real-world analyses than in the benchmark study [12,13]. In respect to iBRAF/iMEK treatment in the metastatic setting, adjuvant treatment resulted in a slightly lower incidence of AEs, with pyrexia in 35.4% of patients, gastrointestinal symptoms in 37%, and cutaneous manifestation in 14% of patients [13]. None of the above-mentioned AEs have been reported to correlate with response to therapy or better outcome.

Although evidence remains limited, several case series and retrospective analyses suggest that immune-related adverse events (irAEs) may occur during iBRAF and iMEK therapy, likely reflecting the immunomodulatory effects of MAPK pathway inhibition [14,15]. In some reports, these events have been associated with durable responses, as observed in patients treated with immunotherapy, supporting a potential link between immune activation and clinical benefit. Indeed, a retrospective analysis of a cohort of 78 patients identified vitiligo, uveitis, erythema nodosum and keratosis sicca as irAE of iBRAF treatments and demonstrated an association between these irAEs and a higher patient PFS. This association was independent of LDH level and disease burden [14]. iBRAF/iMEK therapy may exert an immunomodulatory effect in patients with melanoma, promoting the release of pro-inflammatory cytokines such as interferon (IFN)-γ in the tumor microenvironment, increasing T-cell infiltration and stimulating their cytotoxic activity [16,17,18,19]. Moreover, analyses of primary or metastatic tissue samples that differed for the presence or absence of the *BRAF* mutation, indicated a diverse immune microenvironment, with a significant decrease in CD8+ T cells and an increase in B cells and CD4+ T cells in *BRAF*-mutated tumors [20].

Therefore, the aim of this study was to analyze in a cohort of patients with melanoma the occurrence of irAEs and possible indications that the onset of an irAE could be considered a biomarker of response to iBRAF/iMEK therapy. We also analyzed whether any clinical feature or circulating immune cell pattern could also indicate an immunomodulatory effect of the iBRAF/iMEK treatment.

## 2. Materials and Methods

Patients. This study was conducted according to the Good Clinical Practice Guidelines and the Declaration of Helsinki. This study was approved by the Institutional Review Boards of Istituto Dermopatico dell’Immacolata (IDI)-IRCCS (ID #407/1, 2013). All patients enrolled in this study provided written informed consent. Patients with unresectable metastatic melanoma, stage IIIc/IIId or IV based on American Joint Committee on Cancer (AJCC, version 8) staging [21], enrolled at IDI-IRCCS from 2009 to 2020, were included in this study. For the patients undergoing flow cytometry analysis, peripheral blood samples were collected before therapy (T0), after the first (T1) and second (T2) month of therapy, and after four (T4) additional months of treatment. Therefore, the T4 samples correspond to a six-month therapy. Demographic and clinicopathological features of patients are illustrated in Table 1 and Table 2. Patients were treated with iBRAF (dabrafenib or vemurafenib) either as monotherapy or in combination with iMEK (cobimetinib or trametinib). Dabrafenib was administered at a dose of 150 mg twice daily (BID), while vemurafenib was given at a dose of 960 mg BID. Cobimetinib was administered at a dose of 60 mg once daily for 21 consecutive days followed by a 7-day treatment break, whereas trametinib was administered at a dose of 2 mg once daily. Only one patient was treated with cobimetinib as monotherapy. Patients underwent physical examination and assessment of biochemical parameters monthly, whereas clinical objective response was assessed radiologically with computed tomography scans approximately every 12 weeks after treatment initiation. AE evaluation was done accordingly to Common Terminology Criteria for Adverse Events (CTCAE) version 5.0 [22]. Autoimmune AEs were considered irAE.

Flow cytometry analysis. Whole blood samples were collected into vacutainer sodium citrate tubes (cat. no. 367704, BD Biosciences, Plymouth, UK) and peripheral blood mononuclear cells (PBMCs) were isolated by Ficoll gradient centrifugation (GE Healthcare, Little Chalfont, UK). PBMCs were stained with fluorochrome-conjugated monoclonal antibodies directed against human proteins. Specifically, we used two antibody panels: panel 1 contained CXCR5 Alexa488 (BD Biosciences) (1:100), CRTh2-PE (Miltenyi Biotech, Bergisch Gladbach, Germany) (1:150), CD161-PE Dazzle594 (BioLegend, San Diego, CA, USA) (1:50), CD4-BB700 (Beckman Coulter, Brea, CA, USA) (1:100), CD56-PC7 (BioLegend) (1:120), CXCR3-APC Alexa647 (BioLegend) (1:40), CD8-APC Alexa700 (Beckman Coulter) (1:120), TCRγδ-APC Vio770 (Miltenyi Biotech) (1:100), CCR6-BV421 (BioLegend) (1:30), CD3-BV605 (BD) (1:30), PD-1-BV650 (BD) (1:30), CD69-BV785 (BD) (1:80); panel 2 contained CD4-FITC (Miltenyi Biotech) (1:100), CD3-ECD (Beckman Coulter) (1:100), CD127-APC Alexa700 (Beckman Coulter) (1:200), CD19-APC Vio770 (Miltenyi Biotech) (1:100), CD25-BV421 (BioLegend) (1:60).

Both panels included the LIVE/DEAD™ Fixable Aqua Dead Cell Stain Kit (Invitrogen, Thermo Fisher Scientific, Carlsbad, CA, USA) (1:200) to discriminate and exclude dead cells from the analysis. Samples were stained for 30 min at 4 °C, washed with PBS, acquired on a CytoFLEX flow cytometer (Beckman Coulter) and analyzed using FlowJo software, version 10.3.0. All cell populations were analyzed within alive cells, excluding debris and doublets.

Gating strategy for discrimination of different cell populations was performed as previously reported [23] and described in Supplementary Figure S1. Briefly, CD4 (CD3^+^, CD4^+^); CD8 (CD3^+^, CD8^+^); CD8-MAIT (CD3^+^, CD8^+^, CD161^high^); NK cells (CD3^−^, CD56^dim^); NK bright cells (CD3^−^, CD56^high^); γδ T cells (CD3^+^, TCR-γδ^+^); B cells (CD3^−^, CD19^+^). For CD4 T cells we discriminate: Th1 cells (CD3^+^, CD4^+^, CRTH2^−^, CXCR3^+^, CCR6^−^); Th2 cells (CD3^+^, CD4^+^, CRTH2^+^, CXCR3^−^, CCR6^−^, CXCR5^−^); Th17 cells (CD3^+^, CD4^+^, CRTH2^−^, CXCR3^−^, CD161^+^, CCR6^+^, CXCR5^−^); Th1/17 cells (CD3^+^, CD4^+^, CRTH2^−^, CXCR3^+^, CD161^+^, CCR6^+^, CXCR5^−^); Thf cells (CD3^+^, CD4^+^, CRTH2^−^, CXCR3^−^, CD161^−^, CCR6^−^, CXCR5^+^); Treg cells (CD3^+^, CD4^+^, CD127^−^, CD25^high^).

Statistical analysis. Categorical variables (i.e., sex, localization of primary, anatomic site, presence of ulceration, metastatic stage, serum LDH levels, type of drug, line of treatment, toxicity (occurrence yes/no, type) were described as numbers and percentages, and continuous variables (i.e., age at primary melanoma, age at iBRAF/iMEK therapy, Breslow thickness), as mean and standard deviation (SD) or standard error of the mean (SEM), and median and interquartile range (IQR). Patients with toxicity and patients without toxicity were compared in subgroups according to metastatic stage, serum LDH levels, type of drug, line of treatment, using the Fisher’s exact test. Age at iBRAF + iMEK therapy was compared between patients with and without occurrence of toxicity using the Mann–Whitney U test. PFS curves were estimated with the Kaplan–Meier method. The log-rank test was used to compare the PFS in patients with occurrence of toxicity and patients without toxicity. The crude hazard ratio and 95% confidence were estimated with the Cox proportional hazards model.

Statistical analyses were performed using Stata software, release 17 (StataCorp LLC, College Station, TX, USA) and GraphPad Prism 9.0.

## 3. Results

### 3.1. Clinical Characteristics of the Enrolled Patients

For this study, 158 patients mainly affected by cutaneous melanoma (131), 1 by mucosal melanoma and 26 by melanoma of unknown origin, were considered (Table 1). The mean age was 58 years old and male patients were slightly more represented than female patients (94 versus 64). The primary melanoma was present mainly in the trunk (53.2%), with a median Breslow thickness of 3 mm (IQR = 2–5 mm) and 54% of the cutaneous melanoma was ulcerated. At therapy beginning, the mean age was higher (62 years). Metastatic stage varied from M1a to M1d, serum LDH was normal in 37% of patients, elevated in 32%, and no data were available for 31% of patients (Table 2).

The majority of patients received iBRAF/iMEK therapy as a first-line treatment, and the most commonly used drugs were the combination of dabrafenib and trametinib (74.7%), followed by the combination of vemurafenib and cobimetinib (12%) (Table 2).

### 3.2. iBRAF/iMEK-Mediated Toxicity in Patients with Melanoma 

In our patient cohort, toxicity occurred in 101 patients (64%), and patients could have experienced multiple toxicities. Among these patients, approximately half of them experienced cutaneous toxicity, while irAEs were rare and observed in only three patients (Table 3).

The three patients with irAE had vitiligo, parapsoriasis, and uveitis, respectively.

Besides cutaneous AEs, other highly represented AEs were fever (45 patients), asthenia (34 patients), and gastrointestinal type (27 patients). Liver, bone marrow, and skeletal muscle were also affected (17, 15, and 8 patients, respectively) (Supplementary Table S1). No differences were found in terms of age at iBRAF/iMEK therapy initiation, metastatic stage, serum LDH levels, type of drug, or line of treatment among patients with or without occurrence of toxicity (Table 4). Among the patients who received iBRAF/iMEK therapy as a second-line treatment, 67% (18/27) received ICI immunotherapy. The washout interval between the two therapies was 30–40 days.

**Figure 1:**
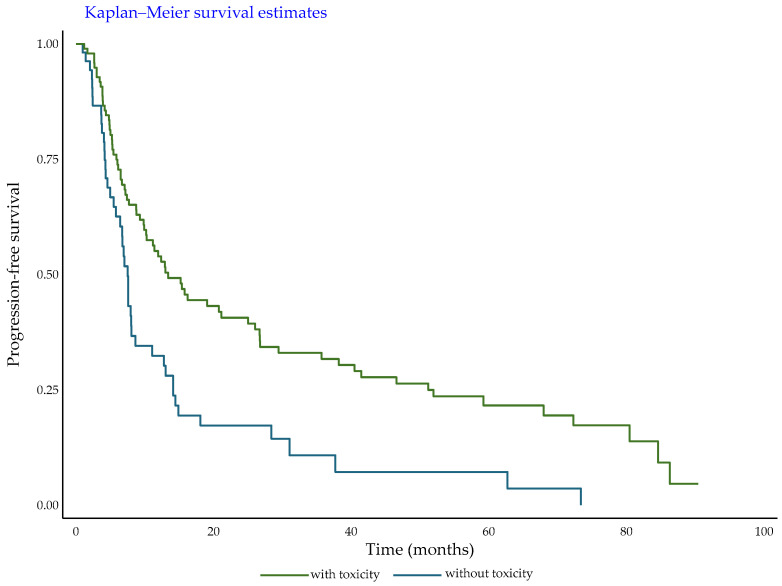
Patient PFS from iBRAF/iMEK therapy initiation.

From therapy initiation, the mean follow-up time for PFS was 19.0 months (ranging from 16 days to 90.4 months) with a median PFS of 8.7 months. Patients with occurrence of toxicity showed a median PFS of 10.9 months, whereas patients without occurrence of toxicity showed a median PFS of 6.7 months (*p* < 0.001) with a reduction of half the risk of progression for patients with occurrence of toxicity in comparison with patients without occurrence of toxicity (*crude* HR = 0.51, 95%CI: 0.34–0.74, *p* < 0.0001) (Figure 1).

### 3.3. Analyses of the Circulating Immune Cell Subsets in iBRAF/iMEK-Treated Patients with irAEs

To better understand whether iBRAF/iMEK therapy could induce an immunomodulatory effect that may lead to the onset of an irAE, we investigated the presence of diverse circulating immune cells in the patients with melanoma who had irAEs during iBRAF/iMEK treatment. We had collected peripheral blood samples and extracted the PBMCs before therapy (T0) and at the subsequent first (T1), second (T2), and sixth month (T4) of treatment of the patients who developed vitiligo or uveitis. Samples of three patients treated with the same drug combination (dabrafenib + trametinib) as a first-line treatment but who did not have any irAEs were used as controls. Samples for the patient who developed parapsoriasis were not available, but some clinical features were analyzed anyway. As reported in Table 5, five patients were male, one was female, with a median age of 60.8 years, staging before therapy either M1c or M1d. The primary melanoma was localized at the trunk for five patients and in the head/neck region for one patient. The three patients who developed an irAE had a worse outcome, with a PFS of 6, 11, and 12 months, respectively, and similar months of OS (6, 12, and 13 months, respectively). The irAE uveitis was developed after 4 months of therapy, vitiligo after 8 months, and parapsoriasis after only 2 months of treatment. We report the overall response (OR) after six months of therapy, since it corresponds to the T4 sample.

**Figure 2:**
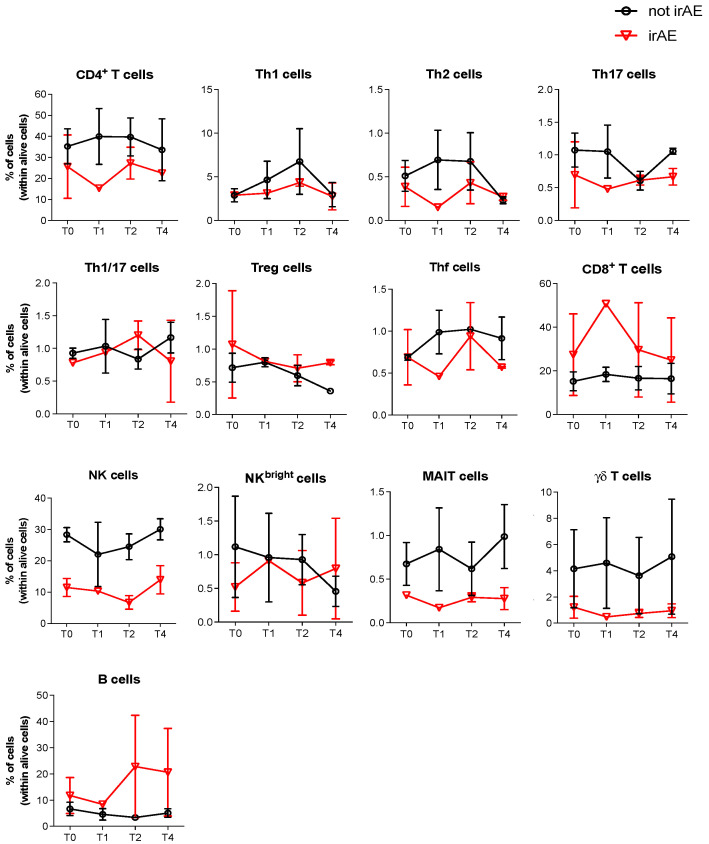
Cytofluorimetric analyses of the immune cell subsets present in the peripheral blood of patients who developed an irAE during therapy (red line, irAE) and in patients who did not develop an irAE (black line, not irAE). Blood samples were taken before therapy (T0), after one month (T1) or two months (T2) of treatment, and after six months of treatment (T4).

Then, we analyzed by cytofluorimetric assays the frequency of the different immune cell subsets in the peripheral blood of the two patients who developed an irAE (uveitis and vitiligo) and also of the three matched control patients. As shown in Figure 2, no differences in the subsets of circulating immune cells were observed in the patients who developed an irAE compared with the control patients. However, a trend toward a lower frequency of circulating T helper (Th)17 and natural killer (NK) cells (Figure 2) and a higher frequency of circulating T regulatory (Treg) cells was observed in the patients with irAEs (Figure 2).

**Figure 3:**
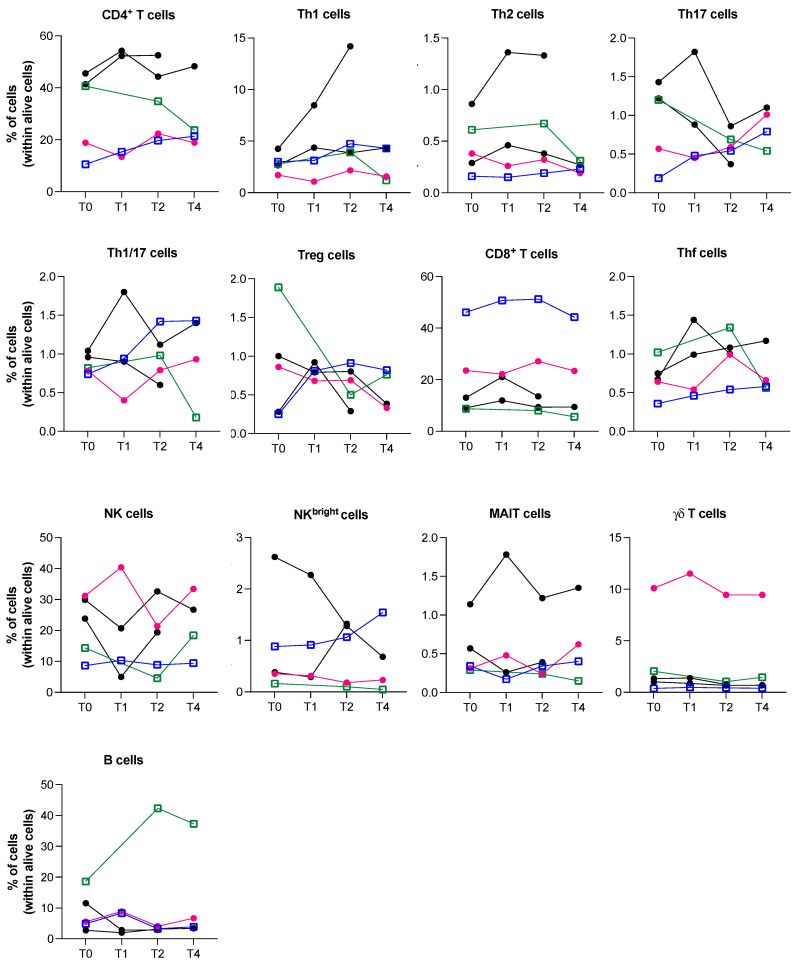
Cytofluorimetric analyses of the immune cell subsets present in the peripheral blood of the five patients who were treated with iBRAF/iMEK therapy. Pink line is associated with the unique patient who achieved a CR as BOR, the green line with the patient who had uveitis as an irAE and a PD after six months of treatment, the blue line with the patient who had vitiligo as an irAE. The lines corresponding to the other two patients are in black. The lines corresponding to the two patients who had irAEs are indicated by the empty squares.

To better understand whether iBRAF/iMEK treatment could influence the frequency of specific immune cell subsets in patient circulation, we analyzed immune cell subsets as the average value of all the five patients under study (Figure 3).

Interestingly, we did not observe major differences in the amount of immune cell subsets in each patient before therapy initiation, with the exception of a trend toward increasing the amount of circulating T helper follicular (Thf) cells within the first two months of therapeutic treatment. Then, the Thf cell amount either decreased or maintained the achieved level. No differences were seen for the one patient who achieved a CR as BOR, except for a higher amount of γδ T cell frequency that was already evident at baseline, before therapy (Figure 3, pink line). The patient who had uveitis as an irAE, and a PD after six months of treatment, showed a reduction in CD4^+^ T cells, Th1/Th17 cells and Treg cells with progression (Figure 3, green line).

**Figure 4:**
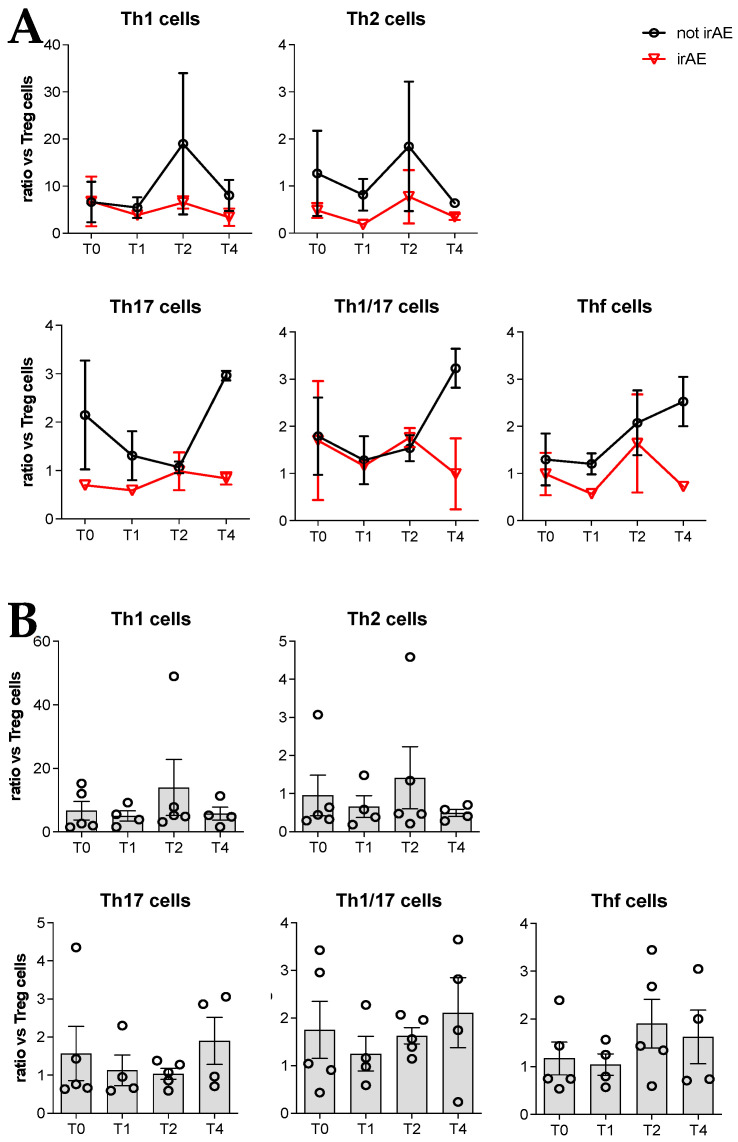
Cytofluorimetric analyses of the immune cell subsets present in the peripheral blood of patients (**A**) who developed an irAE during therapy (irAE) and in patients who did not develop an irAE (not irAE) or (**B**) of the patients altogether. Results are reported as the ratio between different T helper immune cell subtypes and Treg cells.

Considering that Treg cells are greatly involved in the onset of autoimmune reactions, we also analyzed the ratio of different effector cells to Treg cell frequency (Figure 4). We found that the ratio of Th1/Th17 cells and of Thf cells to Treg cells were lower in the patients who developed irAEs compared with patients who did not (Figure 4A). When the five patients were analyzed altogether no difference was observed in the ratio with Treg cells (Figure 4B).

**Figure 5:**
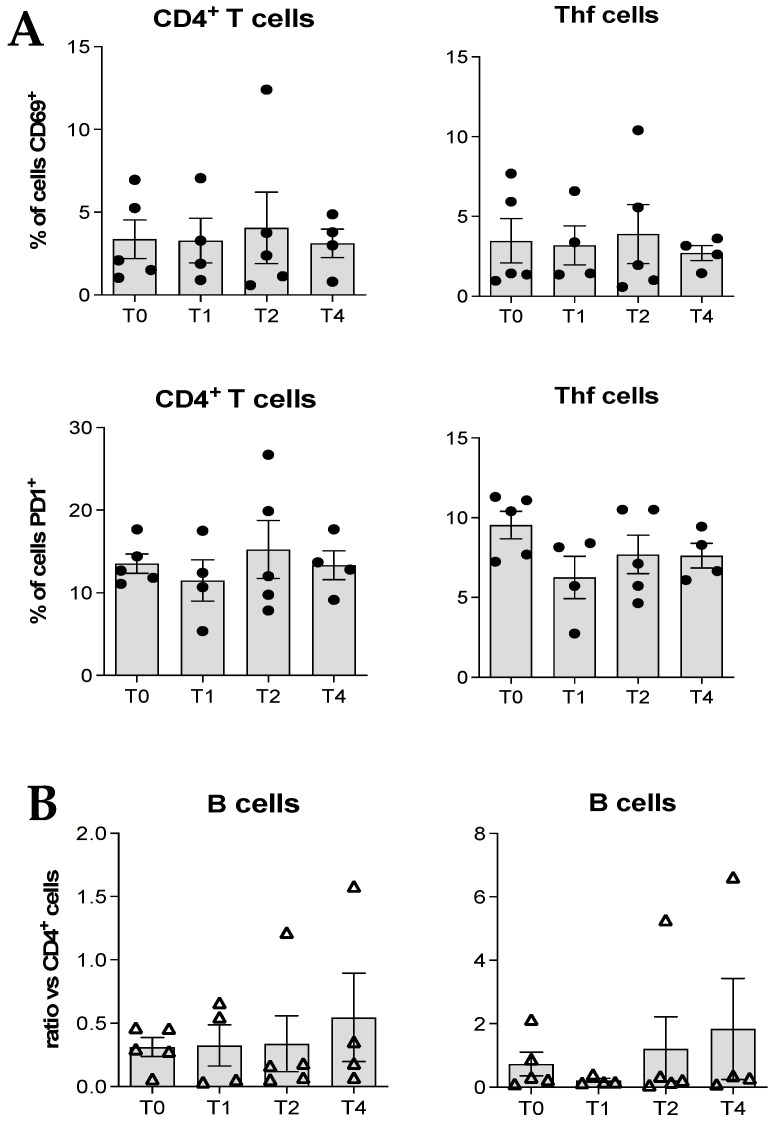
Cytofluorimetric analyses of (**A**) the activation and exhaustion status for CD4^+^ T cells and Thf cells, based on CD69 and PD1 expression, respectively, as well as (**B**) the B cells to CD4^+^ T cells ratio.

We also analyzed the activation status of the CD4^+^ T cells and of the circulating Thf cells by considering the expression of the CD69 marker and found no changes during the therapy administration (Figure 5A). Same data were obtained for the expression of PD1 considered as an exhaustion marker (Figure 5A). No difference during the therapy course was observed for the ratio between B cells and CD4^+^ cells (Figure 5B).

**Figure 6:**
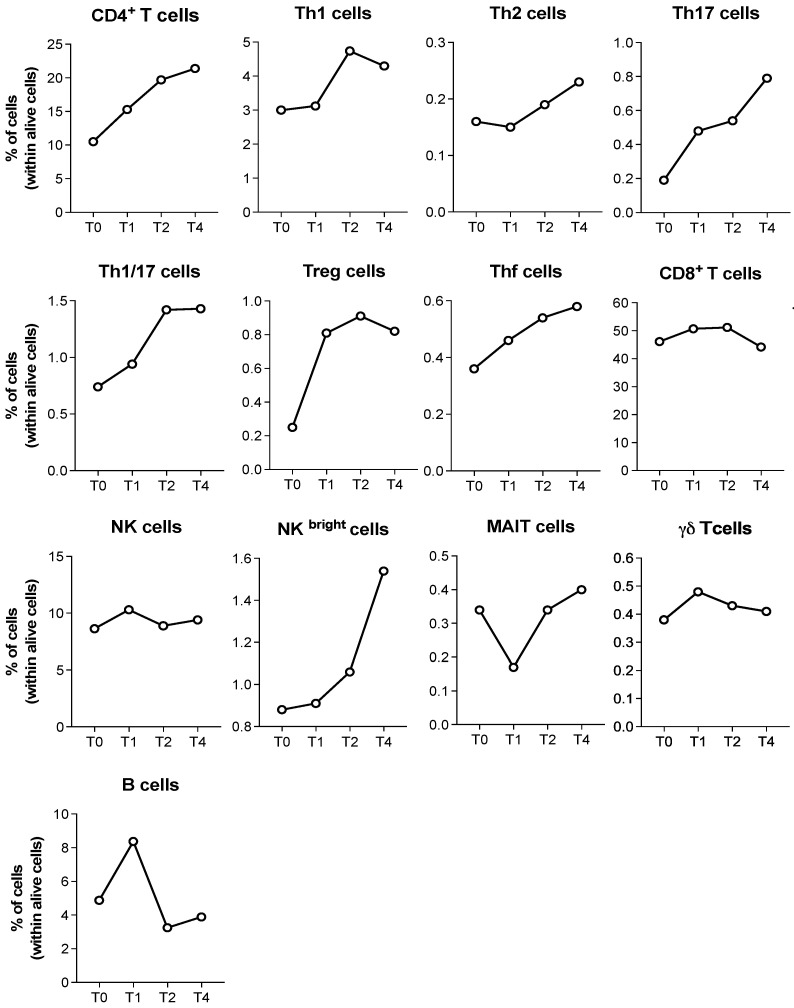
Cytofluorimetric analyses of the immune cell subsets present in the peripheral blood of the patient who developed vitiligo as an irAE during iBRAF/iMEK therapy.

Finally, we specifically analyzed the frequency of immune cell subtypes in the patient who developed vitiligo as an irAE. We found a trend toward an increased frequency of several circulating CD4^+^ T cell subsets, including Th1, Th2, Th17, Th1/17, Thf, Treg cells, and NK^bright^ cells, during the course of therapy (Figure 6).

## 4. Discussion

The aim of our study was to evaluate the frequency of irAEs in patients with melanoma treated with iBRAF/iMEK therapy and to establish whether the onset of an irAE could be considered a biomarker of response to treatment. Among the 101 patients who developed an AE in our cohort, we found that irAEs occurred in 3 patients (3%). This occurrence is lower than the 12.8% previously observed by Ben-Betzalel et al. [14] in a cohort of 78 patients and could be due to the different iBRAF drugs used. In fact, the majority of their patients were treated with vemurafenib alone, whereas the majority of our patients were treated with the combination of dabrafenib and trametinib. Notably, those authors underlined that they did not perform a proper clinical evaluation of the developed irAE and, therefore, we hypothesize that there is the possibility of an overestimation of irAEs in their results. Ben-Betzalel et al. also demonstrated an association between irAEs and a higher PFS. Considering the clinical features of the three patients who developed an irAE in our cohort, we could not find any indication that correlate the irAE with a response to treatment or a better outcome. However, we found out that the development of toxicity is associated with a longer PFS, with a reduction of half the risk of progression for patients with occurrence of toxicity in comparison with patients without occurrence of toxicity.

Interestingly, of the 78-patient cohort of the Ben-Betzalel et al. study, 4 patients developed vitiligo and 4 uveitis, both involving skin or eye areas rich in melanocyte antigens. In the three patients who experienced irAEs in our cohort, one case of vitiligo and one of uveitis was seen, further supporting the possibility of a cross-reactive autoimmune response against normal melanocytes. Indeed, it was recently demonstrated that iBRAF/iMEK target therapy modulates tumor cell immunopeptidome in vitro, rendering tumor cells attackable by the immune system [24]. BRAF targeting can also sensitize melanoma cells to cytotoxic T cells by upregulating the mannose-6-phosphate receptor [18].

Despite various indications about the possible involvement of immune system modulation in the clinical response to iBRAF/iMEK, to our knowledge the circulating immune cell subsets in iBRAF/iMEK-treated patients with melanoma have not yet been investigated. In the present study, we initially analyzed the different subsets of immune cells by comparing patients who did or did not develop irAEs during treatment. We did not find any difference in immune cell subsets between the two patient sets. Patients who developed irAEs tended to have a lower frequency of circulating Th17 and NK cells, whereas circulating Treg cells tended to be more represented. The ratio between Thf cells and Treg lymphocytes was also lower. However, due to the small number of cases that we could examine, further studies are required to confirm these observations.

To better understand the immune modulation due to iBRAF/iMEK treatment, the five patients were analyzed altogether. Interestingly, a trend towards the increase in the frequency of Thf cells was observed in the first two months of target therapy. Circulating Thf cells did not show any differences in their activation or exhaustion status. Thf cells are a specialized subset of CD4^+^ T cells that connect the cellular and humoral immunity, as they lead to B cell proliferation and activation, as well as to antibody production [25]. Thf cells greatly contribute to the development of autoimmune diseases, and elevated levels of circulating Thf cells have been observed in patients with systemic lupus erythematosus and rheumatoid arthritis, which is associated with disease severity and autoantibody levels [26]. A previous study described a case of delayed autoimmune-like reaction to BRAF/MEK inhibition, with the circulating increment of various cytokines, such as IL-23, IL-6, IL-10, IL-17A/F, IL-1β, and IL-21, highlighting a possible role for Th/Thf activation in the irAE pathogenesis [27]. Even if in that study the authors did not analyze the circulating immune cells, their results and ours suggest the possibility of an increment of circulating Thf cells is specifically due to iBRAF/iMEK target therapy. It was previously reported that patients with high B lymphocyte infiltration into the tumor had a decreased MAPK activity and increased expression of immunosuppressive markers [28]. Therefore, there is the possibility that increased levels of circulating Thf cells represent a feedback result of iBRAF/iMEK treatment aiming at restoring a correct B cell homeostasis and anti-tumor activity. Moreover, a high B cell signature was associated with poor survival in patients treated with iBRAF-iMEK [28], highlighting the necessity to more deeply investigate the relationship between circulating Thf cells, B cell activity, and iBRAF/iMEK treatment responses in patients with melanoma. It is interesting to note that an opposite behavior has been observed in patients with melanoma treated with immune checkpoint inhibitors, where an increment in B cells was associated with a favorable outcome [29,30]. Additional studies are required to clarify the significance of these aspects.

Nevertheless, our descriptive data shed light on a iBRAF/iMEK-dependent immune regulation that should be taken into account when the novel therapeutic approaches that combine iBRAF/iMEK with immune checkpoint inhibition are considered [31,32,33,34].

Clinical trials are currently performed to assess the most effective sequential combination of immunotherapy with checkpoint inhibitors and iBRAF/iMEK target treatment. The results of the SECOMBIT and DREAMseq trials established that immunotherapy with both anti-PD-1 and anti-CTLA-4 (nivolumab/ipilimumab) inhibitors should be considered as the standard of care for first-line treatment, also for patients with BRAFV600-mutant melanoma [31,34]. The phase III DREAMseq trial, where patients were randomly assigned to receive either nivolumab/ipilimumab or dabrafenib/trametinib as first-line therapy, led to an early clinically significant end point: the 2-year OS for those starting nivolumab/ipilimumab was 71.8% versus 51.5% (*p* = 0.010) for those starting iBRAF/iMEK [34]. Other reasons for using immunotherapy first in the combination sequence were the response rate (88% of responses to nivolumab/ipilimumab versus < 50% with dabrafenib/trametinib), and the efficacy of BRAF target therapy in the second-line setting. Indeed, immune checkpoint inhibitors appeared less effective after progression with dabrafenib/trametinib than as a first-line treatment [31]. Moreover, real-world experiences confirmed the superiority in prescribing first nivolumab/ipilimumab over first iBRAF/iMEK [35,36]. However, whether selected patients may obtain additional benefit from a short course of target therapy before immune checkpoint treatment remained an open question [31].

CTLA-4 axis regulates Thf cells and inhibits B cell responses [37]. Thus, there is a possibility that immunotherapy with anti-CTLA-4 antibodies can lead to heightened B cell responses with a simultaneous expansion of the Thf cell population [37]. Based on the results we present here, subsequent iBRAF/iMEK therapy could further increase the frequency of circulating Thf cells. Therefore, this aspect requires deeper investigation to understand its possible significance in the development of an effective anti-tumor therapeutic protocol.

Differently from what happens with ICI immunotherapy, where we and others reported a vitiligo onset in the 10–28% of patients with melanoma treated for metastatic disease [38,39], de novo development of vitiligo as an irAE in metastatic melanoma patients treated with iBRAF/iMEK is rarely reported [40]. Besides our patient, and the four reported in Ben-Betzalel et al., only a few more cases have been described in the literature [41]. This makes it extremely difficult to demonstrate that the onset of irAEs is due to iBRAF/iMEK treatment. In fact, vitiligo is seen also in naïve-treatment patients with melanoma [42]. The only patient who developed vitiligo in our cohort had an initial response to iBRAF/iMEK treatment, lasting 11 months, then the disease rapidly progressed. Subsequent treatment with the immune checkpoint inhibitor pembrolizumab was ineffective and the patient died soon after. In this case, vitiligo development after eight months of treatment was not associated with a positive outcome. This patient had the tendency to increase the frequency of CD4^+^, Th1, Th2, Th17, Treg, and NK^bright^ cells in the blood circulation, whereas the frequency of MAIT cells was not changed during the course of therapy. Conversely, comparing the frequency of circulating immune cells in patients who developed vitiligo during checkpoint inhibitor immunotherapy, we found a downregulation of Th17, Treg, NK^bright^, and MAIT cells in the blood [23]. Therefore, there is the possibility that what was observed in the patient treated with iBRAF/iMEK is more associated with a disease progression than with vitiligo onset.

We found that the ratio between Th17 and Treg cells tended to be lower in the patients who developed irAEs in respect to controls during treatment with iBRAF/iMEK. Examining circulating immune cells in patients with melanoma who developed vitiligo during immune checkpoint treatment, we found that the decrease in circulating Th17 cells was accompanied by an increase in this T cell subset in the vitiligo skin lesion [23]. Unfortunately, we could not verify this issue in the patient who developed vitiligo following iBRAF/iMEK treatment.

The main restraints of this study were the retrospective observational nature and the low number of biological samples available for immunological analyses. The term irAE was originally developed for immunotherapy and later applied to target therapy in a limited number of studies. Considering the difficulty of clearly defining an adverse event as immune-related versus inflammatory, we propose using the term “immune-mediated adverse event” (im-AE) instead of irAE in the future. Our study clearly indicates that irAEs are rare in iBRAF/iMEK-treated patients and hardly related to response to treatment. On the other hand, considering the irAEs altogether, there is the possibility that they could be related to a longer PFS. Our study may also suggest that the immunomodulatory effect of iBRAF/iMEK treatment could be mediated by an alteration of circulating Thf cell levels.

## 5. Conclusions

Unlike previous studies on immunotherapy with ICIs, our study shows that it is currently impossible to correlate the onset of an irAE with a better outcome in patients with metastatic melanoma treated with iBRAF/iMEK target therapy. Conversely, the onset of toxicity is associated with longer PFS. Our study highlights that iBRAF/iMEK therapy modulates circulating immune cell subsets and may increase circulating Thf lymphocytes. Additional studies are needed to determine the importance of this increase in Thf cells for an effective response to therapy and improved patient outcomes.

## Figures and Tables

**Table 1:** Characteristics of the 158 patients enrolled.

Characteristics	N.	%
Age at primary melanoma, y		
Mean (SD)	58.1 (15.5)	
Median (IQR)	58 (47–70)	
Sex		
Male	94	59.5
Female	64	40.5
Localization of primary		
Skin	131	82.9
* Nodular*	*46*	*35.1*
* Superficial spreading*	*70*	*53.4*
* Other*	*15*	*11.5*
Mucosal	1	0.6
Unknown	26	16.5
Anatomic site		
Head/neck	17	10.8
Upper limbs	17	10.8
Trunk	84	53.2
Lower limbs	24	15.2
Unknown	16	10.1
Breslow thickness, mm *		
Mean (SD)	4.3 (5.5)	
Median (IQR)	3 (2–5)	
Ulceration *		
Present	71	54.2
Absent	47	35.9
Unknown	13	9.9

SD, standard deviation; IQR, interquartile range. * Only for cutaneous melanoma.

**Table 2:** Patient characteristics at the start of iBRAF/iMEK therapy.

Characteristics	N.	%
Age at iBRAF/iMEK therapy, years		
Mean (SD)	61.7 (14.3)	
Median (IQR)	62 (52–73)	
Metastasis stage		
M1a	41	25.9
M1b	24	15.2
M1c	56	35.4
M1d	37	23.4
Serum LDH		
Normal	58	36.7
Elevated	51	32.3
Unknown	49	31.0
Drug		
D + T	118	74.7
D	8	5.1
V + C	19	12.0
V	12	7.6
C	1	0.6

SD, standard deviation; IQR, interquartile range; LDH, lactate dehydrogenase; D, dabrafenib; T, trametinib; V, vemurafenib; C, cobimetinib.

**Table 3:** Patients with occurrence of toxicity after iBRAF/iMEK therapy, by toxicity type.

	N.	%
Toxicity after iBRAF/iMEK		
No	57	36.1%
Yes	101	63.9%
Types of toxicity		
Cutaneous only	12	11.9%
Non-cutaneous only	50	49.5%
Cutaneous and non-cutaneous	39	38.6%
irAE *	*3*	*3.0%*

* Including 1 vitiligo, 1 parapsoriasis, 1 uveitis.

**Table 4:** Characteristics of patients and occurrence of toxicity.

	Toxicity		No Toxicity		
		N = 101		N = 57	
Characteristics:	N. *	%	N. *	%	*p* Value **
Age at iBRAF + iMEK therapy, y					
Mean (SD)	61.9 (13.8)		61.5 (14.9)		
Median (Range)	63 (52–72)		61 (52–72)		0.981 ***
Sex					
Male	59	58.4	35	61.4	
Female	42	41.6	22	38.6	0.739
Metastatic stage					
1a	26	25.7	15	26.3	
1b	15	14.9	9	15.8	
1c	35	34.6	21	36.8	
1d	25	24.8	12	21.1	0.964
Serum LDH					
Normal	42	41.6	16	28.1	
Elevated	32	31.7	19	33.3	
Unknown	27	26.7	22	38.6	0.182
iBRAF/iMEK line of treatment					
First-line	87	86.1	42	76.4	
Second-line	14	13.9	13	23.6	0.129

SD, standard deviation; LDH, lactate dehydrogenase. * Totals may vary because of missing values; ** Fisher’s exact test; *** Mann–Whitney U test.

**Table 5:** Clinical characteristic of the three patients who had irAEs with iBRAF/iMEK therapy and of the control patients.

Patient	BRAF57	BRAF84	BRAF16	BRAF11	BRAF27	BRAF73
**Sex ^a^**	M	M	F	M	M	M
**Age ^b^**	57	55	52	71	75	55
**Stage ^c^**	M1c	M1d	M1d	M1c	M1d	M1c
**Drug**	Dabrafenib + Trametinib	Dabrafenib + Trametinib	Dabrafenib + Trametinib	Dabrafenib + Trametinib	Dabrafenib + Trametinib	Dabrafenib + Trametinib
**Concomitant** **medications**	None	Anti-hypertensive drugs	Oral steroids	Anti-hypertensive drugs; ASA; statin; glibomet	Anti-hypertensive drugs; ASA; statin	None
**Prior autoimmune disease**	No	No	No	No	No	No
**Line of therapy**	1°L	1°L	1°L	1°L	1°L	1°L
**Prior ICI** **exposure**	No	No	No	No	No	No
**BOR ^d^**	PR	PR	SD	CR	PR	PR
**OR (6 mo)**	PD	PR	SD	PR	PR	PR
**PFS (mo)**	6	11	12	48	11	14
**OS (mo)**	6	12	13	78	12	20
**Anatomic site**	Trunk	Trunk	Trunk	Head/neck	Trunk	Trunk
**Time to irAE** **onset (mo)**	4	8	2	-	-	-
**irAE**	Uveitis	Vitiligo	Para- psoriasis	-	-	-
**Grading ^e^**	G2	G1	G2	-	-	-
**Management**	Topical treatment with steroid and anti-inflammatory eye drops	Nothing	Topical treatment	-	-	-
**Dose interruption/** **reduction**	Interruption of Trametinib	No	No	-	-	-
**Outcome**	Improvement (G1)	Stable	Stable	-	-	-
**ClinR (2 mo)**	PR	SD	SD	-	-	-
**LDH (U/L)**				-	-	-
**baseline**	-	790 (↑)	1100 (↑)			
**2 mo**	-	632	251			

^a^ M, male; F, female; ^b^ age in years at the time of therapy initiation; ^c^ stage before starting therapy; ^d^ BOR, best overall response according to the RECIST criteria; CR, complete response; PR, partial response; SD, stable disease; PD, progressive disease; OR, overall response after 6 months (mo) of treatment; PFS, progression-free survival; OS, overall survival; anatomic site, site of the primary tumor; time to irAE onset, time of the onset of the irAE indicated as months after therapy initiation; ClinR (2 mo), clinical response at 2 months; Grading ^e^, according to CTCAE v5.0; ICI, immune check-point inhibitor; ASA, acetylsalicylic acid.

## Data Availability

Raw data are available from the corresponding author upon reasonable request.

## Supplementary Materials

The following supporting information can be downloaded at: https://www.mdpi.com/article/10.3390/cancers18071072/s1, Table S1: Description of the toxicity occurred after iBRAF+iMEK therapy; Figure S1: Gating strategy for discrimination of different cell populations.

## Abbreviations

The following abbreviations are used in this manuscript:

MEK	Mitogen-Activated Protein Kinase
PD1	Programmed Death-1 Protein
CTLA-4	Cytotoxic T-Lymphocyte-Associated Protein 4
