# Supplementary material for: Immune-Related Adverse Events in Patients with Melanoma Treated with B-RAF/MEK Target Therapy: Occurrence and Circulating Immune Cell Analysis
Source: Cancers (Basel). 2026 Mar 26;18(7):1072. doi: 10.3390/cancers18071072 (PMC13072210; doi:10.3390/cancers18071072)

cancers MDPI

Supplementary Table S1. Description of the toxicity occurred after iBRAF+Imek therapy

Toxicities	N.
Cutaneous	
rash	10
hyperkeratosis	10
skin cancer	10
panniculitis	8
xerosis	5
erythema	4
prurigo	3
Hand-Foot Syndrome	2
dermatofibroma	1
alopecia	1
vitiligo	1
parapsoriasis	1
not specified	15
Other types	
fever	45
asthenia	34
gastrointestinal	27
hepatic	17
hematologic	15
cardiological	1
neurological	1
ocular	6
skeletal muscle	8
thyroid	1
mucositis	3
dysgeusia	1

Supplementary Figure S1. **Gating strategy for immune cell characterization by polychromatic flow cytometry.** Human PBMCs purified from peripheral blood of melanoma patients were stained with fluorochrome-conjugated specific antibodies to discriminate different immune cell populations, whose names are reported in red. Arrows indicate sequential gating steps, and plots marked with an asterisk were gated on live cells, excluding debris and doublets. The plots show data from one representative patient.

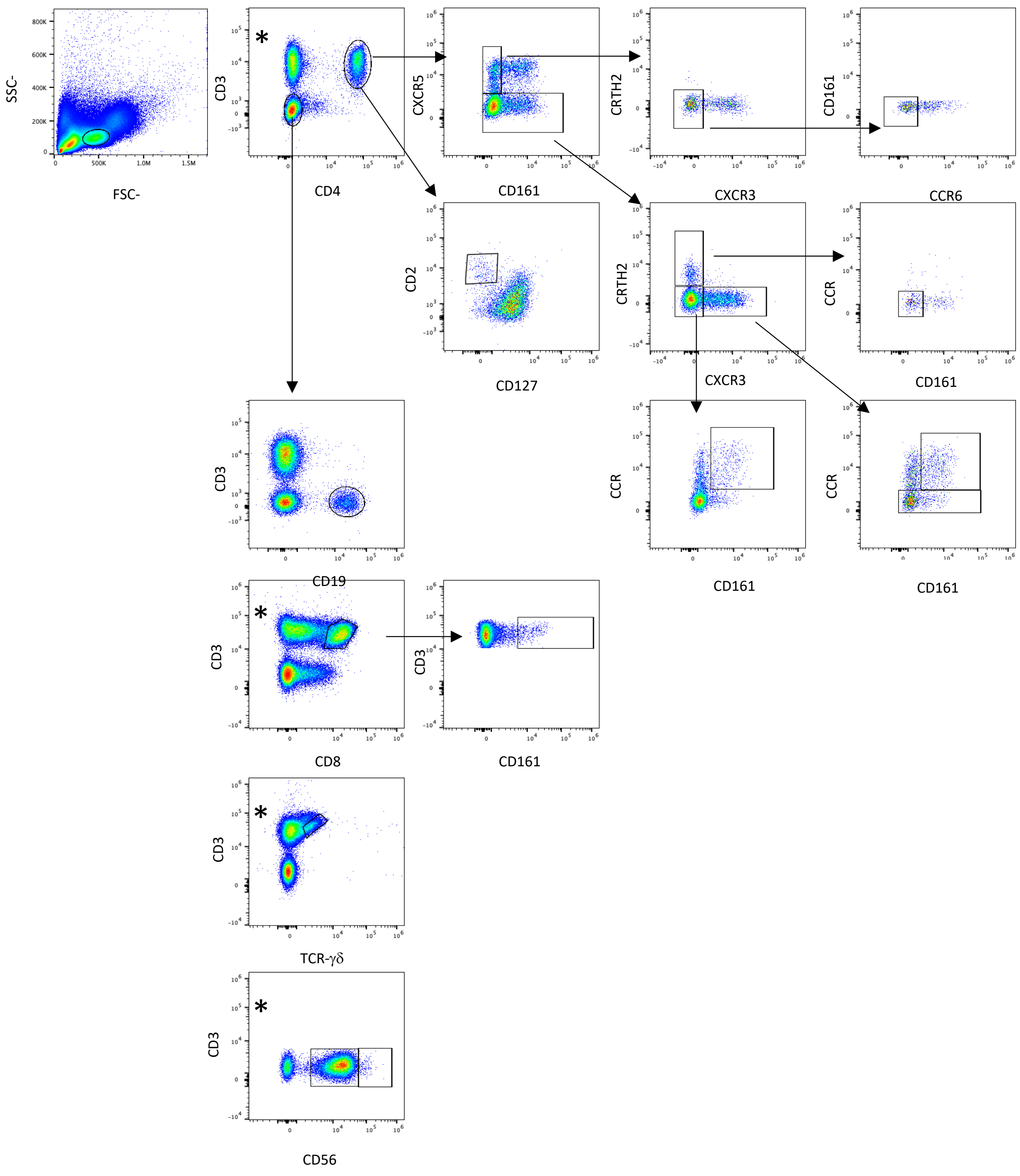

Supplement: Supplementary file 1 [file cancers-18-01072-s001.zip › cancers-4175424-supplementary.pdf]
